# Colorectal Cancer Apoptosis Induced by Dietary δ-Valerobetaine Involves PINK1/Parkin Dependent-Mitophagy and SIRT3

**DOI:** 10.3390/ijms22158117

**Published:** 2021-07-29

**Authors:** Nunzia D’Onofrio, Elisa Martino, Luigi Mele, Antonino Colloca, Martina Maione, Domenico Cautela, Domenico Castaldo, Maria Luisa Balestrieri

**Affiliations:** 1 Department of Precision Medicine, University of Campania Luigi Vanvitelli, Via L. De Crecchio 7, 80138 Napoli, Italy; elisa.martino@unicampania.it (E.M.); antonino.colloca@studenti.unicampania.it (A.C.); martina.maione@unicampania.it (M.M.); marialuisa.balestrieri@unicampania.it (M.L.B.); 2 Department of Experimental Medicine, University of Campania Luigi Vanvitelli, Via Luciano Armanni 5, 80138 Naples, Italy; luigi.mele@unicampania.it; 3 Stazione Sperimentale per le Industrie delle Essenze e dei Derivati dagli Agrumi (SSEA), Azienda Speciale CCIAA di Reggio Calabria, Via G. Tommasini 2, 89125 Reggio Calabria, Italy; dcautela@ssea.it (D.C.); dcastaldo@ssea.it (D.C.); 4 Ministero dello Sviluppo Economico (MiSE), Via Molise 2, 00187 Roma, Italy

**Keywords:** colon cancer, mitophagy, mitochondrial dysfunction, PINK1/Parkin

## Abstract

Understanding the mechanisms of colorectal cancer progression is crucial in the setting of strategies for its prevention. δ-Valerobetaine (δVB) is an emerging dietary metabolite showing cytotoxic activity in colon cancer cells via autophagy and apoptosis. Here, we aimed to deepen current knowledge on the mechanism of δVB-induced colon cancer cell death by investigating the apoptotic cascade in colorectal adenocarcinoma SW480 and SW620 cells and evaluating the molecular players of mitochondrial dysfunction. Results indicated that δVB reduced cell viability in a time-dependent manner, reaching IC50 after 72 h of incubation with δVB 1.5 mM, and caused a G2/M cell cycle arrest with upregulation of cyclin A and cyclin B protein levels. The increased apoptotic cell rate occurred via caspase-3 activation with a concomitant loss in mitochondrial membrane potential and SIRT3 downregulation. Functional studies indicated that δVB activated mitochondrial apoptosis through PINK1/Parkin pathways, as upregulation of PINK1, Parkin, and LC3B protein levels was observed (*p* < 0.0001). Together, these findings support a critical role of PINK1/Parkin-mediated mitophagy in mitochondrial dysfunction and apoptosis induced by δVB in SW480 and SW620 colon cancer cells.

## 1. Introduction

Colorectal cancer (CRC) is the third most diagnosed cancer worldwide in 2020 with an estimated 1.8 million new cases [1]. Over 930,000 deaths from CRC were projected to occur in 2020, making it the second leading cause of cancer-related death [1]. Epidemiologic research approximates that half of colon cancer risk is preventable by modifiable risk factors, including diet [2]. The dietary δ-valerobetaine (δVB) (N,N,N-trimethyl-5-aminovaleric acid) is an emerging naturally occurring metabolite with antioxidant, anti-inflammatory and anti-cancer activities, displaying cytotoxic effects in colon cancer and head and neck squamous cell carcinomas [3,4,5,6]. In head and neck squamous cell carcinomas, δVB caused a severe inhibition of cell proliferation together with induction of apoptosis and cell cycle arrest in the G2/M phase [6]. δVB also determined an accumulation of mitochondrial reactive oxygen species (ROS) and influenced the expression and activity of SIRT1. Apoptosis and cell cycle arrest attenuation upon SIRT1 gene silencing supported the potential role of δVB as an epi-nutrient, thus acting as an anticancer agent through the modulation of SIRT1 [6]. In human colon cancer LoVo cells, δVB inhibited cell viability and induced cell cycle modulation, causing necrosis and a massive intracellular, extracellular and mitochondrial ROS production responsible for SIRT6 activation and apoptotic cell death [5]. The apoptotic cell death mediated by δVB was accompanied by the activation of autophagy, as demonstrated by the inhibition of the death rate when LoVo cells were treated with the lysosomal inhibitor chloroquine, thus indicating that autophagy is not merely a general stress response, but it drives apoptosis [5]. Moreover, the cytotoxic activity of δVB could also be ascribed to changes in l-carnitine metabolism, thus interfering with the β-oxidation process and taking part in the stress-induced cell death mechanism [7].

Altered metabolism is a hallmark of colon cancer, as cancer cells use several metabolic substrates to sustain the production of ATP and intermediate precursors for nucleotide, fatty acid and amino acid synthesis required for cell proliferation and tumor progression [8]. In this regard, mitochondrial biogenesis involves the coordinated expression of peroxisome proliferator-activated receptor-coactivator-1 (PGC-1), the major regulator of this process, which in turn activates the nuclear and mitochondrial transcription factors and induces the expression of sirtuin 3 (SIRT3). SIRT3, the major mitochondrial deacetylase, is considered a fidelity protein for mitochondrial function as it activates enzymes that regulate mitochondrial metabolism and the cellular response to oxidative stress [9]. Although SIRT3 was described as a tumor promoter in some types of cancers by shifting cellular metabolism toward increased glycolysis, in other types of cancers SIRT3 acts a tumor suppressor by modulating ROS and limiting the oxidative damage in cellular components [9]. In vitro studies showed that silencing SIRT3 in colon cancer cells resulted in an increase in oxidative burden, changes of the mitochondrial biogenesis, inhibition of cell proliferation and increased activation of apoptosis, thus consequently sensitizing cells to cytotoxic treatments [10,11,12]. Moreover, reversing the dysregulated metabolism of glucose and fatty acid in colon cancer by dihydrotanshinone I (DHTS) has been shown to be linked to lower levels of SIRT3 gene expression within a metabolic reprogramming occurring through the PTEN/AKT/RHEB/MTOR/HIF1α signal pathway [13]. Clinical observations indicated that in colon cancer patients, the survival rate was 64.6% among patients with high SIRT3 expressions and 88.6% among patients with low SIRT3 expressions (log-rank P = 0.016) [12].

The availability of nutrients and reprogrammed metabolic pathways used by cancer cells regulates autophagy and mitophagy. Mitophagy, the mitochondria-selective autophagy, is fundamental for a healthy network of functioning mitochondria and the clearance of dysfunctional or obsolete mitochondria, which are recognized by the autophagy machinery and degraded by the lysosome [14]. The mitochondrial PTEN-induced kinase-1 (PINK1) and the E3 ubiquitin ligase Parkin [15] represent a protective mechanism by which cancer cells oppose the onset of mitochondrial apoptosis [16]. However, defective mitophagy can influence diverse cellular pathways that are characteristics of tumors and an adequate mitochondrial clearance is likely to contribute to tumor suppression at numerous molecular levels [17]. Several studies reported that mitophagy can not only protect cells from death, but they also displayed pro-death functions [18].

Despite recent evidence describing the ROS-mediated apoptotic cell death induced by δVB in LoVo colorectal cancer cells [5], the complete pathway(s) through which this bioactive metabolite triggers mitochondrial dysfunction and apoptosis is not fully elucidated. Moreover, in order to strengthen the potential of targeted redox-modulating strategies in the prevention of colorectal cancer, it is critical to broaden the investigation to a wider panel of colon cancer cell lines, reflecting the diverse stages of this malignancy. To this end, the present study aimed to dissect the molecular mechanism of the apoptotic death triggered by δVB in SW480 (Dukes’ type B colon cancer) and SW620 (Dukes’ type C colon cancer) cells and deepen the understanding of the molecular players of mitochondrial dysfunction.

## 2. Results

### 2.1. Inhibition of Colon Cancer Proliferation

**Figure 1:**
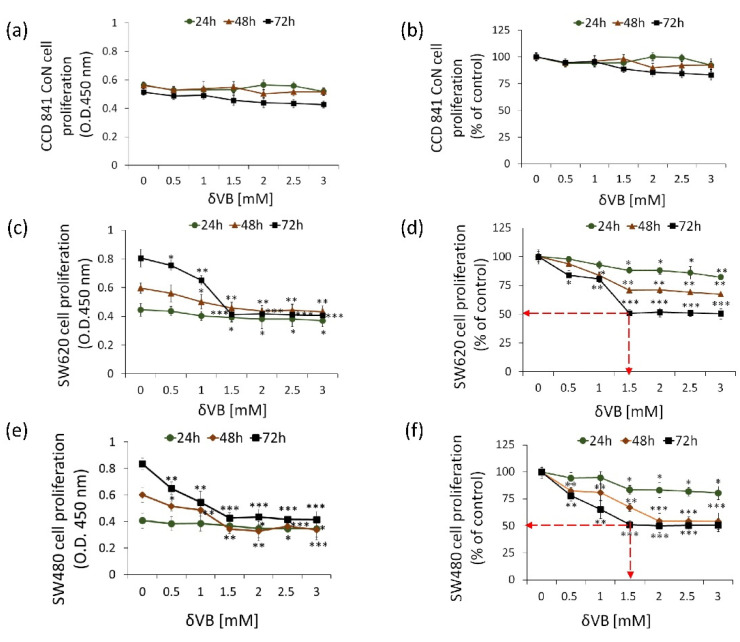
Effects of δVB on SW620 and SW480 cell proliferation. (**a**,**b**) CCD 841 CoN, (**c**,**d**) SW620 and (**e**,**f**) SW480 cells were treated with increasing concentrations of δVB (0–3 mM) for 24, 48 and 72 h. The IC50 in SW620 and SW480 was determined at 72 h incubation with 1.5 mM δVB and represented as red dotted arrows. Control cells were grown in medium containing the same volume of HBSS-10 mM Hepes. Cell proliferation inhibition was assessed using Cell Counting Kit-8 assay (Donjindo Molecular Technologies, Tokyo, Japan). Values represent the mean ± SD or the % of control of four independent experiments. * *p* < 0.05 vs. Ctr; ** *p* < 0.01 vs. Ctr; *** *p* < 0.001 vs. Ctr.

δVB was tested on human colorectal adenocarcinoma cell line SW480 (Dukes’ type B colon cancer) and SW620 (metastasis, Dukes’ type C), and on normal colon CCD 841 CoN cells. Treatments with δVB (up to 3 mM) for 72 h weakly affected non-malignant CCD 841 CoN cell proliferation (17.0 ± 5.0% vs. Ctr) (Figure 1a,b).

On the other hand, significant cytotoxic effects were detected when SW480 and SW620 cells were treated with increasing concentrations of δVB (0–3 mM) for 24, 48 and 72 h (Figure 1). Results showed a time- and dose-dependent capability of δVB to selectively inhibit the proliferation of SW480 and SW620 cells reaching the IC50 value at 1.5 mM after 72 h of δVB treatment (*p* < 0.001 vs. Ctr) (Figure 1c–f). This δVB dose (1.5 mM) was chosen to perform the subsequent experiments of this study.

### 2.2. Cancer Cell Cycle Progression

**Figure 2:**
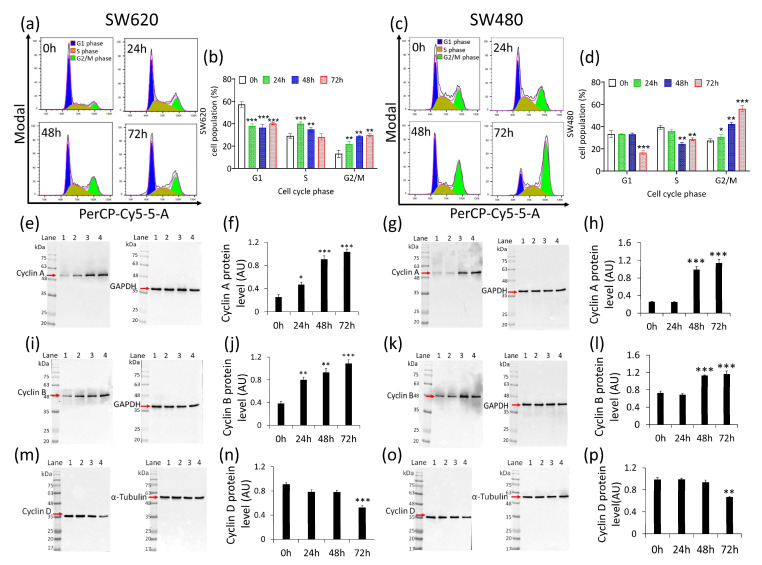
Effects of δVB cell cycle. Representative cell cycle analysis and average of (**a**,**b**) SW620 and (**c**,**d**) SW480 cell cycle distribution. Cells were treated with δVB (1.5 mM) for 24, 48 and 72 h. Cell cycle distribution was evaluated by flow cytometry collecting PI fluorescence as FL3-A (linear scale) and analyzed by ModFIT software (Verity Software House, Becton Dickinson, Topsham, ME, USA). For each sample, at least 10,000 events were acquired. Representative full-length blots of Western blotting analysis of cyclin A (**e**–**h**), cyclin B1 (**i**–**l**) and cyclin D (**m**–**p**) in SW620 and SW480 cells, respectively. Lane 1 = 0 h; lane 2= 24 h, lane 3 = 48 h, lane 4 = 72 h. Before lane 1; molecular weight markers (G266, Applied Biological Materials Inc., Richmond, BC, Canada). Protein expression was calculated, after normalization with internal control (α-tubulin or GAPDH), with ImageJ software and results expressed as arbitrary units (AU). * *p* < 0.05 vs. Ctr; ** *p* < 0.01 vs. Ctr; *** *p* < 0.001 vs. Ctr.

Cell cycle analysis of SW620 cells showed the capability of δVB to increase cells in S phase after 24 and 48 h treatment (40.0% ± 1.6% and 35.0% ± 1.6%, respectively, vs. 29.1% ± 2.0% of Ctr) (*p* < 0.001), and in G2/M checkpoint after 48 and 72 h (28.8% ± 0.8% and 29.8% ± 1.6%, respectively, vs. 13.1% ± 3.1% of Ctr) (*p* < 0.01) (Figure 2a,b). 

In SW480 cells, δVB treatment led to a time-dependent arrest in G2/M cell cycle phase. Indeed, the G2/M population increased from 27.4% ± 1.7% in the control to 56.0% ± 2.8% after 72 h of exposure to δVB (*p* < 0.001) (Figure 2c,d). In addition, the effect of δVB on cell cycle regulation was accompanied by an accumulation of cyclin A (Figure 2e–h) and cyclin B (Figure 2i–l) protein levels (*p* < 0.001 vs. Ctr). Conversely, δVB downregulated cyclin D at 72 h of incubation (Figure 2m–p) (*p* < 0.001 vs. Ctr). Finally, no effects on cell cycle modulation were observed on colon CCD 841 CoN cells after 24, 48 and 72 h treatment with 1.5 mM δVB (Supplementary Materials Figure S1).

### 2.3. Caspase-3 Activation

**Figure 3:**
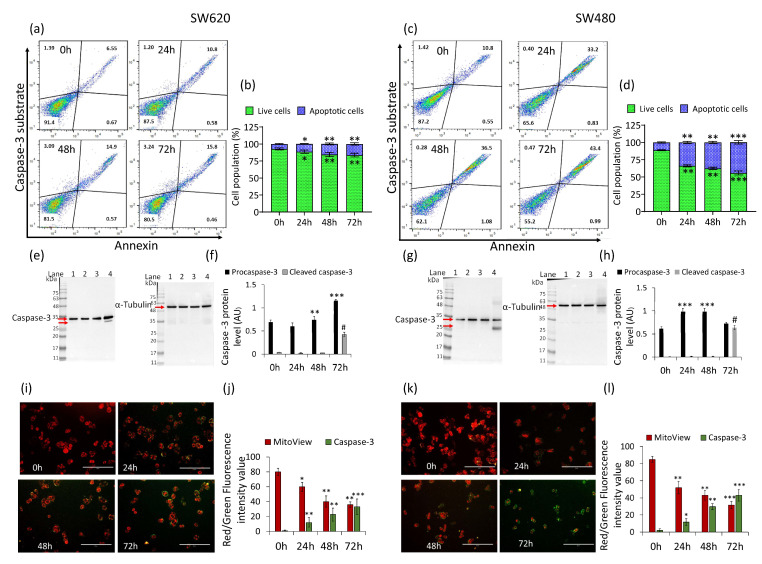
Effects of δVB on caspase-3 activation. Representative dot plots and analyses of caspase-3 activation by reporting annexin CF 594 and caspase-3 substrate on (**a**,**b**) SW620 and (**c**,**d**) SW480 cells treated with δVB (1.5 mM) at 24, 48 and 72 h. Cell population was assessed by flow cytometry and results expressed as % of live or apoptotic population with mean ± SD of n = 3 experiments. At least 10,000 events were acquired. Representative full-length blots of Western blotting analysis of caspase-3 in (**e**,**f**) SW620 and (**g**,**h**) SW480 cells. Lane 1 = 0 h; lane 2 = 24 h; lane 3 = 48 h; lane 4 = 72 h. Before lane 1, molecular weight markers (G266, Applied Biological Materials Inc., Richmond, BC, Canada). Protein expression was calculated, after normalization with α-tubulin as internal control, with ImageJ software and results expressed as arbitrary units (AU). Representative images and analysis of Nucview 488 and Mitoview 633 staining of (**i**,**j**) SW620 and (**k**,**l**) SW480 cells analyzed by fluorescence microscopy. Results expressed as red and green fluorescence intensity value. Scale bar: 100 μm. * *p* < 0.05 vs. Ctr; ** *p* < 0.01 vs. Ctr; *** *p* < 0.001 vs. Ctr; # *p*< 0.0001 vs. Ctr.

In SW620 cells, δVB treatment increased caspase-3 activity (% of apoptotic cells) in a time-dependent manner, reaching the maximum activity rate at 72 h (16.3% ± 1.8% vs. 7.2% ± 0.8% of Ctr) (*p* < 0.01) (Figure 3a,b). 

A more consistent effect was reached in SW480 cells (Figure 3c,d), showing an increase of caspase-3 activity up to 44.4% ± 2.5% after 72 h compared to untreated cells (11.3% ± 0.8%) (*p* < 0.001). Western blot analysis showed the ability of δVB to induce the inactive (procaspase) and active (caspase) forms in both SW620 and SW480 cells (Figure 3e–h) (*p* < 0.0001 vs. Ctr). Moreover, in SW620 and SW480 cells, δVB induced caspase-3 activation accompanied by a depletion of mitochondrial polarization (*p* < 0.001 vs. Ctr) (Figure 3i–l). 

### 2.4. Mitochondrial Perturbation and SIRT3 Downregulation

**Figure 4:**
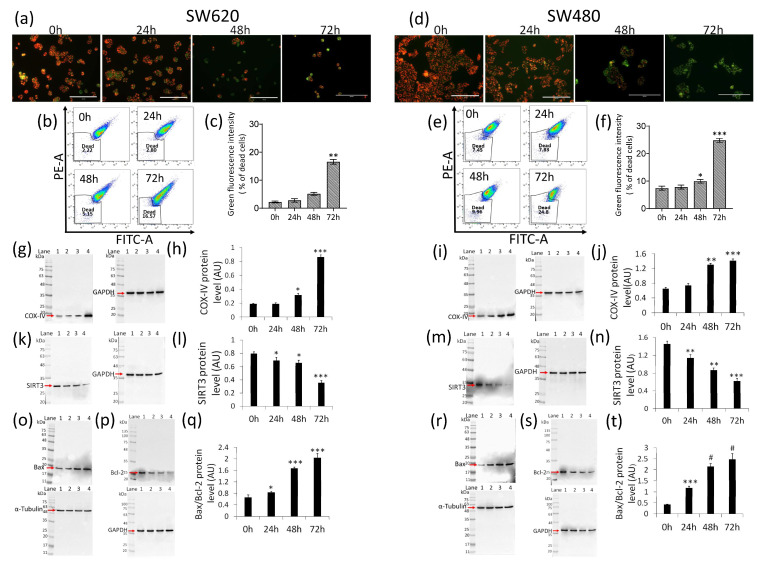
δVB effects on mitochondrial status. Representative images, dot plots and analysis of mitochondrial membrane potential assessed using JC-1 probe, which allows us to detect membrane depolarization through a concomitant decrease in the aggregate signal (red) and accumulation of monomeric species (green), of (**a**–**c**) SW620 and (**d**–**f**) SW480 cells. Cells were treated with δVB (1.5 mM) for 24, 48 and 72 h. Results, expressed as green fluorescence intensity, were reported as % of dead cells. Scale bar: 200 μm. Representative immunoblotting images and analyses of (**g**–**j**) COX-IV, (**k**–**n**) SIRT3 and (**o**–**t**) Bax/Bcl-2 protein levels in SW620 and SW480 cells. Lane 1 = 0 h; lane 2 = 24 h; lane 3 = 48 h; lane 4 = 72 h. Before lane 1, molecular weight markers (G266, Applied Biological Materials Inc., Richmond, BC, Canada). Protein expression was calculated, after normalization with internal control (α-tubulin or GAPDH), with ImageJ software and results expressed as arbitrary units (AU). * *p* < 0.05 vs. Ctr; ** *p* < 0.01 vs. Ctr; *** *p* < 0.001 vs. Ctr; # *p*< 0.0001 vs. Ctr.

The assessment of the mitochondrial polarization rank with JC-1 fluorescent probe indicated a marked time-dependent increase of the SW620 cell population (death rate expressed as % of green fluorescent) (16.6% ± 1.0% vs. 2.2% ± 0.3% of Ctr) during treatment with δVB (*p* < 0.01) (Figure 4a–c). 

A notable death rate was also detected in SW480 cell population after 72 h of treatment (24.8% ± 0.7% vs. 7.4% ± 0.8% of Ctr) (*p* < 0.001) (Figure 4d–f). Moreover, δVB was found to upregulate COX-IV, the enzyme of the mitochondrial electron transport chain (Figure 4g–j) (*p* < 0.001 vs. Ctr), and downregulate the mitochondrial SIRT3 (Figure 4k–n) (*p* < 0.001 vs. Ctr). Lastly, in SW620 cells, δVB mediated the simultaneous upregulation of the pro-apoptotic Bax (Figure 4o) and downregulation of the anti-apoptotic Bcl-2 expression levels (Figure 4p), thus resulting in an increased Bax/Bcl-2 ratio (Figure 4q) (*p* < 0.001 vs. Ctr). In SW480 cells, the effect of δVB on the increased Bax/Bcl-2 ratio was even more pronounced (Figure 4r–t) (*p* < 0.0001 vs. Ctr).

### 2.5. Activation of Mitophagy

**Figure 5:**
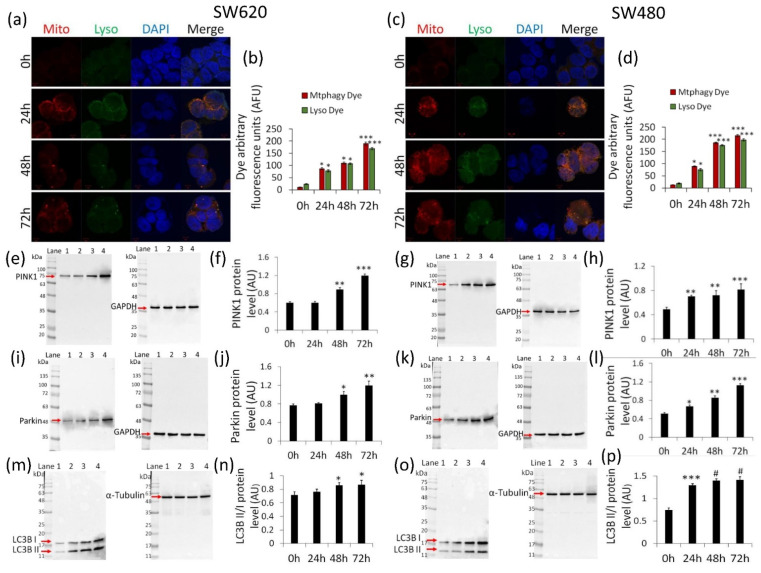
δVB effects on mitophagy. Representative confocal images of mitophagy (red) and lysosome (green) dyes and analysis of (**a**,**b**) SW620 and (**c**,**d**) SW480 cells treated with δVB (1.5 mM) for 24, 48 and 72 h. Nuclei were counterstained with DAPI (blue). Fluorescence intensity analysis was performed by ImageJ software and expressed as arbitrary fluorescence units (AFU) of dye signal ± SD of n = 3 replicates. Scale bar: 5 μm. Representative immunoblotting images and analyses of (**e**–**h**) PINK1, (**i**–**l**) Parkin and (**m**–**p**) LC3B protein levels in SW620 and SW480 cells. Lane 1 = 0 h; lane 2 = 24 h; lane 3 = 48 h; lane 4 = 72 h. Before lane 1, molecular weight markers (G266, Applied Biological Materials Inc., Richmond, BC, Canada). Protein expression was calculated, after normalization with internal control (α-tubulin or GAPDH), with ImageJ software and results expressed as arbitrary units (AU). * *p* < 0.05 vs. Ctr; ** *p* < 0.01 vs. Ctr; *** *p* < 0.001 vs. Ctr; # *p* < 0.0001 vs. Ctr.

We next investigated the possible involvement of mitophagy in cells exposed to δVB. Results indicated a time-dependent increase of mitophagic and lysosomic fluorescence in SW620 cells (Figure 5a,b) (*p* < 0.001 vs. Ctr). 

Likewise, in SW480 cells, δVB mediated a fluorescent accumulation signal (Figure 5c,d) (*p* < 0.001 vs. Ctr). These findings were supported by the upregulation of PINK1 expression (*p* < 0.001 vs. Ctr) (Figure 5e–h) and Parkin protein levels (*p* < 0.001 vs. Ctr) (Figure 5i–l). Finally, evaluation of LC3B, an autophagic player involved in the autophagosome formation, revealed that δVB induced a time-dependent accumulation of LC3BII, the lipidized form of LC3B correlated to membrane autophagosome (Figure 5m–p) (*p* < 0.0001 vs. Ctr).

### 2.6. Apoptosis Induction

**Figure 6:**
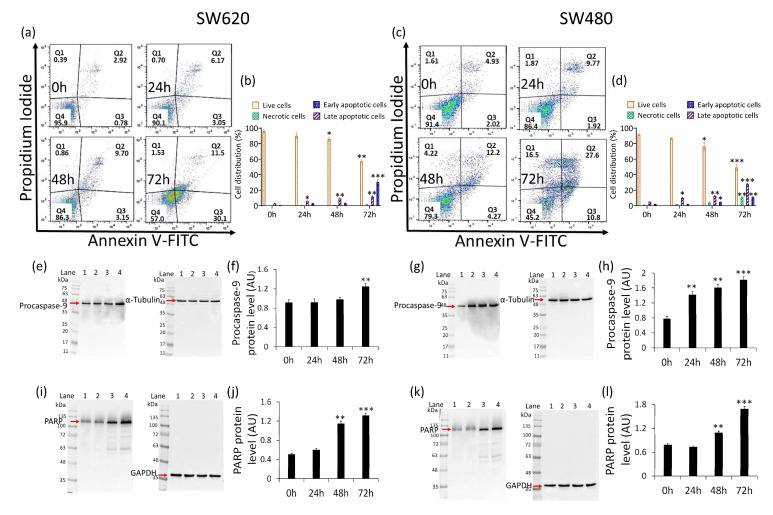
Effects of δVB on colorectal adenocarcinoma cell death. Representative dot plots and analyses of annexin V-FITC and PI-stained (**a**,**b**) SW620 and (**c**,**d**) SW480 cells treated with 1.5 mM δVB for 24, 48 and 72 h. Cell viability/death was assessed by flow cytometry where at least 10,000 events were acquired. Q1: necrotic cells; Q2: late apoptotic cells; Q3: early apoptotic cells; Q4: viable cells. Data are expressed as mean ± SD of n = 3 experiments. Representative full-length blots of Western blotting analysis of procaspase-9 (**e**–**h**) and PARP (**i**–**l**) in SW620 and SW480 cells. Lane 1 = 0 h; lane 2 = 24 h; lane 3 = 48 h; lane 4 = 72 h. Before lane 1, molecular weight markers (G266, Applied Biological Materials Inc., Richmond, BC, Canada). Protein expression was calculated, after normalization with internal control (α-tubulin or GAPDH), with ImageJ software and results expressed as arbitrary units (AU). * *p* < 0.05 vs. Ctr; ** *p* < 0.01 vs. Ctr; *** *p* < 0.001 vs. Ctr.

The measurement of annexin V/PI expression revealed that the cytotoxicity and mitophagy induced by δVB was accompanied by apoptosis. In detail, a time-dependent reduction of live cells (57.1% ± 2.1% vs. 96.0% ± 2.1% of Ctr at 72 h) (*p* < 0.01) and a concomitant increase of the early apoptotic population (30.5% ± 2.9% vs. 0.81% ± 0.04% of Ctr) were observed in SW620 cells (*p* < 0.001) (Figure 6a,b). 

Instead, in SW480 cells, a decrease in live population (49.1% ± 4.0% vs. 91.6% ± 1.2% of Ctr) (*p* < 0.001) was accompanied by a rise in early (10.8% ± 0.9% vs. 2.0% ± 0.2% of Ctr) (*p* < 0.01) and late apoptotic cells (27.7% ± 1.3% vs. 5.1% ± 0.3% of Ctr) (*p* < 0.001), along with necrosis accumulation (10.8% ± 1.2% vs. 1.0% ± 0.1% of Ctr) (*p* < 0.01) (Figure 6c,d). At the same time, exposure of SW620 and SW480 to δVB upregulated procaspase-9 and poly (ADP-ribose) polymerase (PARP) expression levels (Figure 6) (*p* < 0.001). On the contrary, δVB did not affect apoptotic cell death in CCD 841 CoN cells. Results showed the absence of caspase-3, caspase-9 and PARP modulation in CCD 841 CoN cells exposed to δVB (Supplementary Materials Figure S2).

### 2.7. Mitophagy Inhibition and Apoptosis Attenuation

**Figure 7:**
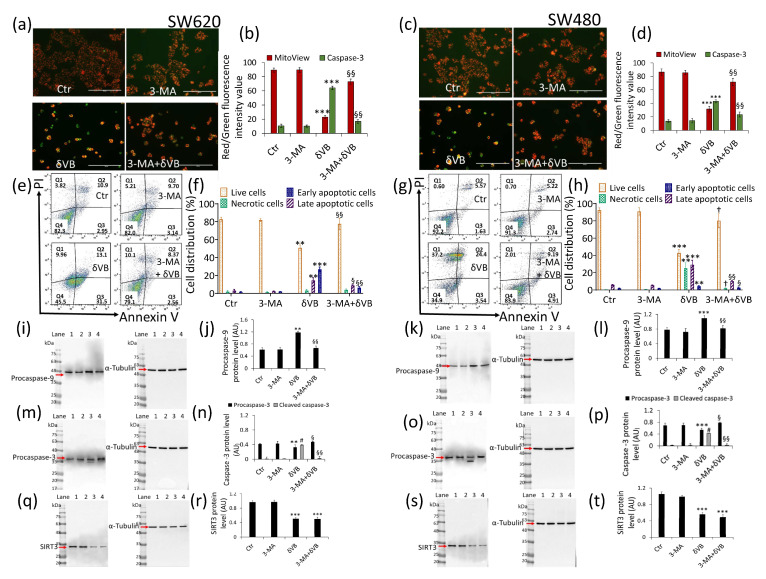
Mitophagy inhibition reduced δVB-mediated apoptosis. Representative images and analysis of Mitoview 633 (red) and Nucview 488 (green) staining of (**a**,**b**) SW620 and (**c**,**d**) SW480 cells. Cells were treated with mitophagy inhibitor 3-MA, δVB (1.5 mM) or combined 3-MA + δVB (1.5 mM) for 72 h, as described under Materials and Methods. Results, expressed as red and green fluorescence intensity, were reported as fluorescence intensity value. Scale bar: 100 μm. Representative dot plots and analyses of annexin V-FITC and PI-stained (**e**,**f**) SW620 and (**g**,**h**) SW480 cells. Cell viability/death was assessed by flow cytometry where at least 10,000 events were acquired. Q1: necrotic cells; Q2: late apoptotic cells; Q3: early apoptotic cells; Q4: viable cells. Data are expressed as mean ± SD of n = 3 experiments. Representative full-length blots of Western blotting analysis of procaspase-9 (**i**–**l**), procaspase-3 (**m**–**p**) and (**q**–**t**) SIRT3 protein expression levels in SW620 and SW480 cells. Lane 1 = Ctr; lane 2 = 3-MA; lane 3 = δVB; lane 4 = 3-MA + δVB. Before lane 1, molecular weight markers (G266, Applied Biological Materials Inc., Richmond, BC, Canada). Protein expression was calculated, after normalization with α-tubulin as internal control, with ImageJ software and results expressed as arbitrary units (AU). ** *p* < 0.01 vs. Ctr; *** *p* < 0.001 vs. Ctr; # *p* < 0.0001 vs. Ctr; § *p* < 0.05 vs. δVB; §§ *p* < 0.01 vs. δVB; † *p* < 0.001 vs. δVB.

Finally, in order to verify the linkage between mitophagy and apoptosis and demonstrate whether the former was a survivor mechanism or if it flowed in the apoptotic death, cell treatments δVB were performed in the presence of the mitophagy inhibitor 3-methyladenine, 3-MA (3-MA + δVB). Results indicated that 3-MA abolished the ability of δVB to promote caspase-3 activation and mitochondrial potential deprivation after 72 h of treatment (Figure 7) (3-MA + δVB vs. δVB, *p* < 0.01). 

Flow cytometry analysis of SW620 cells confirmed that 3-MA was capable of reverting the apoptosis induced by δVB. Indeed, a decreased apoptotic population (10.9% ± 1.2% vs. 44.6% ± 7.2% in δVB) and an increase in viable cells (79.1% ± 6.4% vs. 45.5% ± 9.1% δVB) were observed (*p* < 0.01) (Figure 7e,f). Similar results were observed in SW480 cells, showing that the mitophagy arrest partly reverted the apoptotic cell death induced by δVB treatment, with a decrease in the early and late population (14.1% ± 2.7% vs. 27.94% ± 6.3% δVB) and an increase in the viable counterpart (83.9% ± 7.3% vs. 44.9% ± 8.7% δVB) (*p* < 0.001) (Figure 7g,h). 3-MA also affected the expression levels of caspase-9 and caspase-3 during treatment with δVB. Indeed, in the presence of 3-MA, procaspase-9 protein levels were comparable to control cells (Figure 7i–l), as well as δVB-induced activation of caspase-3 expression (cleaved form) (Figure 7m–p) (*p* < 0.01). These data support the evidence that mitophagy induced by δVB acts as a cell death mechanism by potentiating the apoptotic pathway. Moreover, in the presence of the mitophagy inhibitor, 3-MA, the expression levels of SIRT3 in 3-MA + δVB cells were comparable to treatment with δVB alone, suggesting a role of SIRT3 as an upstream regulator of mitochondrial dysfunction (Figure 7q–t).

## 3. Discussion

**Figure 8:**
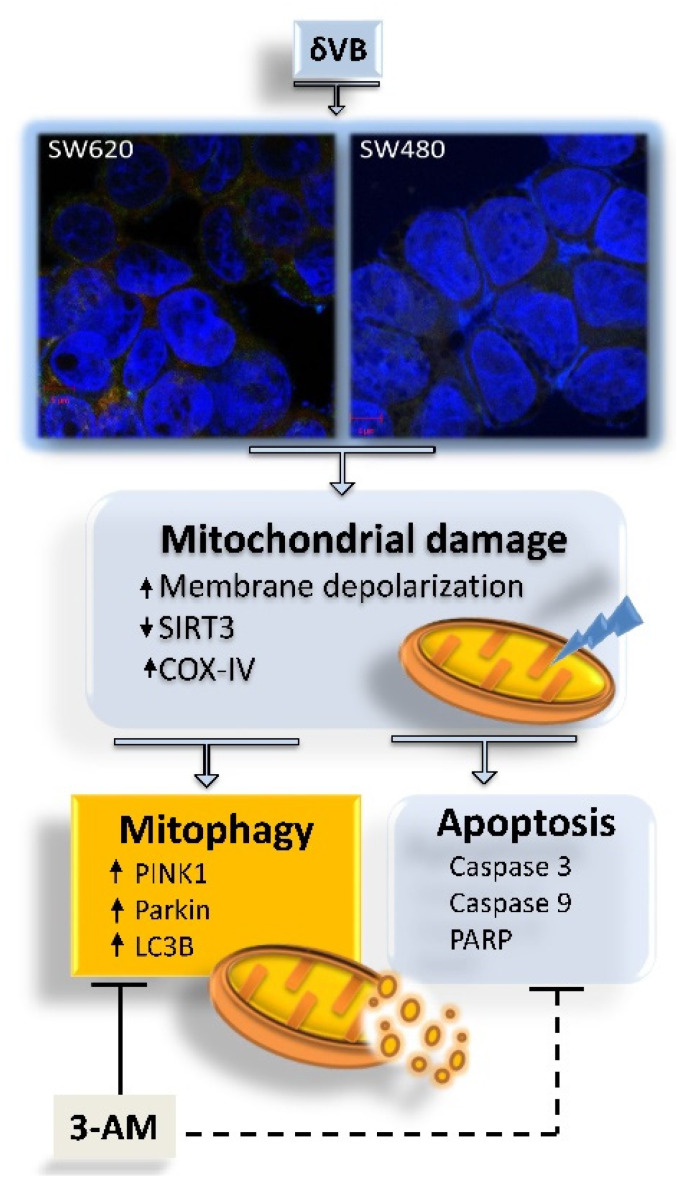
Proposed cellular events leading to colorectal cancer apoptosis induced by δVB. Evidence from the present study suggest that mitochondrial dysfunction and downregulation of SIRT3 induced by δVB fueled the mechanism of mitophagy, which in turn triggered cell death by apoptosis, as evidenced by the inhibition of mitophagy with 3-AM which resulted in the attenuation of intrinsic apoptosis.

In this study, we provide the first evidence that δVB treatment activates mitophagy in SW480 and SW620 colon cancer cells. Treatments with δVB suppress cell viability in a time and dose manner and promote cell cycle arrest in G2/M phase with cyclin A and cyclin B cell accumulation. The δVB-treated cells also exhibit caspase-3 activation, starting from 24 h of treatment, and a mitochondrial membrane depolarization status. Mitochondria are a major intracellular organelle known for energy and ROS production via utilizing macronutrients [19]. Thus, proper mitochondrial dynamics and quality control is required for normal mitochondrial functions. However, little is known about the exact role of mitochondrial quality control in the progression of colorectal cancer [20,21]. This study aimed to deepen the mechanism of δVB-induced apoptosis in SW480 and SW620 colon cancer cells by investigating the possible role of mitophagy in mitochondria dysfunction, known to be induced by δVB in colorectal cancer cells [5]. Mitophagy is a specific autophagy that selectively removes dysfunctional or damaged mitochondria to protect cells from death [22,23]. However, the extent and duration of mitophagy activation is a crucial step for cell survival or death decisions, as abnormal or overactivated mitophagy is toxic to cells. [24]. We found that δVB was able to induce mitophagy, which serves a pro-death role in SW480 and SW620 colon cancer cells, by promoting mitochondrial dysfunction. Mitochondria dysfunction and deregulation of COX-IV, Bax/Bcl-2, and SIRT3 were triggered by δVB. SIRT3 participates in the quality and quantity control of mitochondria. In line with our results showing a time-dependent downregulation of the mitochondrial SIRT3 by δVB, SIRT3 silencing has been shown to reduce motility and colony formation ability and to generally decrease mitochondrial function, ultimately affecting SW620 cell viability [25]. Interestingly, SIRT3 inhibition by the chemotherapeutic agent cisplatin induced acetylation of MTHFD2 (the mitochondrial methylenetetrahydrofolate dehydrogenase/cyclohydrolase) at lysine 88 (K88) and disturbed cellular redox balance in colorectal cancer cells [26]. On the other hand, knockdown of SIRT3 reversed NOS1-induced apoptosis resistance of SW480 and SW620 colon cancer cells [27]. The mitophagy induced by δVB, in turn, triggered apoptotic cell death likely mediated by PINK1/Parkin/LC3B axis, as confirmed by the observations that the chemical abolishing of the mitophagy process by 3-AM treatment significantly alleviated cell apoptosis upon δVB treatment (Figure 8). 

PINK1, a serine/threonine kinase normally found at low levels, accumulates during mitochondrial damage, increased mitochondrial ROS, depolarization and misfolded protein accumulation [28]. The PINK1/Parkin/LC3B axis is known to mediate mitophagy activation in mammalian cells [29]. In particular, Parkin, an E3-ubiquitin ligase, when recruited and activated, drives the ubiquitination of mitochondrial proteins leading to autophagy [30]. Parkin displays a central role in balancing cellular metabolism and its loss led to the transition to aerobic glycolysis (Warburg effect) and excessive production of ROS from damaged mitochondria. Parkin also contributes to metabolic control by p53 by effecting the proteasomal degradation of metabolic enzymes responsible for glycolysis [31]. Overall, most of the proteins involved in the mitophagy processes have been shown to be dysregulated in cancer patients. However, whether they act as a tumor promoter or a tumor suppressor seems to be strongly correlated to cancer subtype and milieu. A downregulation of both PINK1 and Parkin have been observed in colorectal cancer patients [32]. One third of colorectal carcinomas displayed heterozygous loss of PARK2, the Parkin gene, suggesting the notable involvement of this gene in the progression of colorectal cancer [32]. In the present study, δVB enhanced PINK1 and Parkin protein levels in SW480 and SW620 cell lines, supporting the activation of mitophagy as a contributing process in the mitochondria-related apoptosis. These observations are in agreement with previous studies reporting that Parkin modulates apoptotic cell death in response to mitochondrial stressors, as PINK1-induced recruitment of Parkin to mitochondrial membranes sensitizes cells to apoptosis [33]. In line with evidence showing that Parkin-mediated mitophagy is antagonized by the overexpression of pro-survival Bcl-2-family proteins [34], we found that δVB upregulated the Bax/Bcl-2 ratio and exasperated mitochondrial dysfunction by leading mitochondria membrane depolarization, thus supporting the role of δVB as a pro-apoptotic agent of colon cancer cells acting via mitophagy. The comprehension of PINK1/Parkin-related mitophagy signaling in colon cancer is of emerging interest. Recent evidence described the role of Bcl-2-associated athanogen-6 induced BAG6 as a novel receptor in the PINK1/Parkin pathway involved in protein quality control [35]. In BAG6-transfected LoVo cells, the mitophagy process was observed after mitochondrial depolarization and phosphorylation-ubiquitination of mitochondrial proteins [35]. On the contrary, the deletion of this nucleocytoplasmic shuttling protein in SW480 cells abolished mitochondrial mass, mitophagy and PINK1 accumulation [35]. Besides advanced strategies of drug combinations to arrest cancer progression by modulating apoptosis and autophagy [36], naturally occurring bioactive compounds capable of inducing LC3B-related autophagy and apoptotic cancer death are part of cancer prevention strategies [5]. Herein, we reported a notable accumulation of LC3B protein, a key regulator of the autophagic phenomenon, mediated by δVB treatment. Recently, several authors described the LC3B capability in inducing apoptosis in colon cancer cells through the induction of autophagy [37,38,39]. We found that the apoptosis triggered by δVB is mediated by mitophagy, as demonstrated by the changes in the apoptotic cell rate observed in the presence of the mitophagy inhibitor, 3-MA. These data are in line with the current results reporting that the inhibition of mitophagy through PINK1 deletion strongly induced apoptosis arrest in murine colon tumor cells, while PINK1 overexpression increased apoptotic cell death in vivo and suppressed colon tumor xenograft growth [40]. The δVB-mediated accumulation of Parkin is likely related to cyclin D degradation, as shown by the downregulated expression of protein levels appreciated by immunoblotting analysis. Similarly, Yeo et al. reported that Parkin expression was dramatically reduced in glioma cells, while the restoration of its expression promoted G1 phase cell-cycle arrest and mitigated the cell proliferation rate both in vitro and in vivo [41]. Moreover, cell cycle perturbation in Parkin-expressing glioma was accompanied by a considerable reduction in cyclin D1 levels, but not in cyclin E [41]. Overall, the present study suggests that in SW480 and SW620 colorectal cancer cells, the apoptosis induced by dietary δVB involves PINK1/Parkin dependent-mitophagy. Moreover, in line with previous studies [25], our data indicated the role of SIRT3 as a key player in the control of the mitochondrial function in colon cancer cells. The evidence that the inhibition of mitophagy by 3-MA was unable to restore SIRT3 expression levels leads us to hypothesize that the mitochondrial instability exasperated by decreased levels of SIRT3 represents the upstream event triggering the mitophagy. However, it is undeniable that other factors may be involved in this complex and intriguing mechanism. In this regard, further studies are clearly needed to pinpoint the mechanisms through which SIRT3 regulates mitophagy and to better understand how δVB affects SIRT3 activity.

## 4. Materials and Methods

### 4.1. Cell Culture and Treatments

Human colon epithelial CCD 841 CoN cells (CRL-1790) and the human colorectal adenocarcinoma SW480 (CCL-228) and SW620 (CCL-227) were obtained from American Type Culture Collection (ATCC, Manassas, VA, USA). CCD 841 CoN cells were grown in Eagle’s minimum essential medium (EMEM, 30-2003, ATCC, Manassas, VA, USA), while SW480 and SW620 cells were maintained in Leibovitz’s L-15 medium (11-415-064, Gibco, Life Technologies, Carlsbad, CA, USA). Both culture media were supplemented with 100 U/mL penicillin, 100 mg/mL streptomycin and 10% fetal bovine serum (FBS, 10-270-106, Gibco, Life Technologies, Carlsbad, CA, USA). Cells were grown as a monolayer in a humidified incubator, 5% CO_2_, at 37 °C, and the medium was changed 2 times/week. Cells were seeded into multi-well plates the day before treatment to allow cell attachment. δ-Valerobetaine (δVB) synthesis and purification was carried out as previously reported [3]. For cell viability evaluation, treatments were performed by culturing cells in complete medium with increasing δVB concentrations (up to 3 mM), dissolved in Hanks’ balanced salt solution (HBSS)-10 mM Hepes, for a maximum time of 72 h. For further experiments cell lines were maintained with 1.5 mM δVB in complete medium for 0, 24, 48 and 72 h. To selectively inhibit mitophagy, SW480 and SW620 cells were pretreated for 1 h with 5 mM 3-methyladenine (3-MA, M9281, Sigma-Aldrich St. Louis, MO, USA) before treatments with δVB for 72 h (3-MA + δVB). For all cell treatments, control (Ctr) cells were incubated with corresponding volumes of HBSS-10 mM Hepes.

### 4.2. Proliferation Assay

CCD 841 CoN, SW480 and SW620 cells were seeded in 96-well plates at a density of 3 × 10^3^ cells/well. After treatment, cell proliferation was determined using the Cell Counting Kit-8 (CCK-8, Donjindo Molecular Technologies, Inc., Rockville, MD, USA) as previously reported [6]. Proliferation was represented as the mean of the optical density ± SD and as percentage of Ctr. All experiments were performed with n = 4 replicates.

### 4.3. Cell Cycle Evaluation

CCD 841 CoN, SW480 and SW620 cells (9 × 10^4^/well) were seeded in 6-well plates and, after δVB treatment, detached with EDTA-trypsin and stained with BD Cycletest Plus DNA Kit (340242, BD Biosciences, San José, CA, USA) following manufacturer’s instructions, as previously reported [6]. Analysis of data was conducted with the ModFit LT V4.1.7 software (Verity Software House, Becton Dickinson, Topsham, ME, USA).

### 4.4. Apoptotic Cell Death Detection

Viable and apoptotic cells (necrotic, late apoptosis and early apoptosis) were distinguished by the Annexin V Apoptosis detection kit (556547, BD Pharmigen, Franklin Lakes, NJ, USA), as previously reported [6]. Caspase-3 activation and mitochondrial depolarization were monitored with the Nucview 488 and Mitoview 633 Apoptosis Assay kit (30062, Biotium, Fremont, CA, USA). Staining was performed for 15 min at room temperature according to the manufacturer’s instructions. Healthy cells, with an intact mitochondrial membrane potential, were stained with MitoView 633 (red), whereas late apoptotic cells (active caspase-3/7) were stained with NucView 488 (green). Viable and apoptotic cells were imaged on a fluorescence microscope EVOS FL Cell Imaging System (Thermo Scientific, Rockford, IL, USA) and fluorescence intensity was evaluated with ImageJ 1.52n software (National Institutes of Health, Bethesda, MD, USA). Results were expressed as green and red fluorescence intensity value. 

### 4.5. Assessment of Caspase-3/7 Activation

For the caspase-3/7 detection and the visualization of apoptotic nuclear morphology, the dual apoptosis assay with nucview 488 caspase-3 substrate & annexin V kit (30067, Biotium, Fremont, CA, USA) was used according to the manufacturer’s instructions. Briefly, SW480 and SW620 cells were seeded in 6-well plates and treated for 24, 48 and 72 h with 1.5 mM δVB. After treatment and trypsinization, SW480 and SW620 cells were resuspended in 600 μL of 1× binding buffer and incubated in the dark for 30 min at room temperature, with 3 μL of CF594 Annexin and 3 μL of 0.2 mM NucView 488 caspase-3 substrate. The assessment of fluorescence was performed with BD Accuri™ C6 and data were analyzed by FlowJo V10 software. For each sample 20,000 events were recorded.

### 4.6. Mitochondrial Membrane Potential Determination

Mitochondrial membrane potential was assessed using JC-1 staining (MT09, Dojindo Molecular Technologies, Tokyo, Japan), according to the manufacturer’s protocols. After δVB treatments, SW480 and SW620 cells were stained with a 5 μM JC-1 probe at 37 °C for 1 h in the dark. All images were obtained with a fluorescence microscope (EVOS FL Cell Imaging System, Thermo Scientific, Rockford, IL, USA). Subsequently, cells were detached with EDTA-trypsin and fluorescence detected with BD Accuri™ C6. Data were analyzed with FlowJo V10 software and at least 20,000 events were recorded for each sample.

### 4.7. Cell Lysis and Western Blotting Analysis

After treatment, human colorectal adenocarcinoma cell lines were lysed in RIPA lysis buffer (1% NP-40, 0.5% sodium deoxycholate, 0.1% SDS in PBS) containing 10 μg/mL aprotinin, leupeptin and 1 mM phenylmethylsulfonyl fluoride (PMSF). After incubation on ice for 1 h, cell lysates were centrifuged at 10,000× *g* for 15 min at 4 °C and protein content was determined using the Bio-Rad Protein Assay kit (Bio-Rad, Hercules, CA, USA) and compared with a bovine serum albumin (BSA) standard curve. Forty μg of proteins were separated by sodium dodecyl sulfate-polyacrylamide gel electrophoresis (SDS-PAGE) and then transferred to nitrocellulose membranes (Bio-Rad, Hercules, CA, USA). Blots were blocked for 1 h at room temperature (RT) in 1× TBS 1% casein blocker (1610782, Bio-Rad, Hercules, CA, USA) under a gentle shaker. Membranes were then incubated at 4 °C overnight with specific primary antibodies: anti-SIRT3 (1:2000, PA5-86035, Invitrogen, Waltham, Ma, USA), anti-cyclin A2 (1:1000, ab38, Abcam, Cambridge, UK), anti-cyclin B1 (1:1000, 4138, Cell Signaling Technology, Danvers, MA, USA), anti-cyclin D1 (1:1000, ab134175, Abcam, Cambridge, UK), anti-caspase-3 (1:1000, 9662, Cell Signaling Technology, Danvers, MA, USA), anti-caspase-9 (1:1000, 9508, Cell Signaling Technology, Danvers, MA, USA), anti-poly(ADP ribose) polymerase (PARP, 1:1000, ab194586, Abcam, Cambridge, UK), anti-Parkin (1:2000, ab77924, Abcam, Cambridge, UK), anti-Bcl-2 (1:500, E-AB-15522, Elabscience Biotechnology Inc., Houston, TX, USA), anti-COX-IV (1:3000, MA5-15078, Invitrogen, Waltham, MA, USA), anti-BAX (1:500, orb216030, Biorbyt, Cambridge, UK), anti-LC3B (1:1000, ab192890, Abcam, Cambridge, UK), anti-PINK1 (1:500, Y403614, Applied Biological Materials Inc., Richmond, BC, Canada), anti-α-tubulin (1:5000, E-AB-20036, Elabscience Biotechnology Inc., Houston, TX, USA) and anti-glyceraldehyde-3-phosphate dehydrogenase (GAPDH, 1:2000, ab9485, Abcam, Cambridge, UK). After 1 h incubation with HRP-conjugated secondary antibodies (NC GxMu-003-DHRPX and GtxRb-003-DHRPX, ImmunoReagents Inc., Raleigh, NC, USA), the immunocomplexes were examined with the Excellent chemiluminescent substrate kit (E-IR-R301, Elabscience Biotechnology Inc., Houston, TX, USA) and visualized using the ChemiDoc Imaging System with Image Lab 6.0.1 software (Bio-Rad Laboratories, Milan, Italy). After background subtraction, the densities of immunoreactive bands were measured with ImageJ 1.52n software (National Institutes of Health) and expressed as arbitrary units (AU).

### 4.8. Mitophagy Detection

Mitophagy was detected using the Mitophagy Detection Kit (MD01, Dojindo Molecular Technologies, Tokyo, Japan), according to the manufacturer’s instruction. SW480 and SW620 cells were seeded in a 24-well plate containing microscope glass and, after δVB treatments, stained with 100 nM Mtphagy Dye at 37 °C for 30 min. To observe Mtphagy Dye and lysosome co-localization, colon cancer cell lines were subsequently incubated with 1 μM Lyso Dye for an additional 30 min at 37 °C. Cells were then fixed for 20 min with 4% (*v*/*v*) paraformaldehyde solution and permeabilized for 10 min with 0.1% (*v*/*v*) Triton X-100 in PBS. Nuclei were counterstained with 2.5 μg/mL of 4’, 6-diamidino-2-phenylindole (DAPI, Sigma Aldrich, St. Louis, MO, USA) for 7 min. Mitophagy-related fluorescence was assessed with a LSM 700 confocal microscope (Zeiss, Oberkochen, Germany) with a plan apochromat X63 (NA1.4) oil immersion objective. The fluorescence intensity was evaluated with ImageJ 1.52n software (National Institutes of Health, Bethesda, MD, USA) and results expressed as arbitrary fluorescence units (AFU).

### 4.9. Statistical Analysis

All reported data are expressed as mean ± standard deviation (SD). Each experiment was independently conducted in triplicate. IC50 determination and statistical significance were calculated by using GraphPad Prism 9.1.1 software (GraphPad Software Inc., San Diego, CA, USA). The differences between two groups were analyzed using Student’s *t* test, while the differences among three groups were analyzed by one-way ANOVA followed by Tukey post hoc test. A *p*-value smaller than 0.05 was considered to indicate a statistically significant difference. 

## 5. Conclusions

In conclusion, the activation of mitophagy triggered by δVB supports the potential of this dietary nutrient in the prevention of colon cancer [5], broadening the knowledge on the molecular machinery that can be targeted in the setting of promising strategies for colon cancer prevention.

## Data Availability

The data presented in this study are available from the corresponding author upon request.

## Supplementary Materials

The following are available online at https://www.mdpi.com/article/10.3390/ijms22158117/s1.
