# Supplementary material for: Colorectal Cancer Apoptosis Induced by Dietary δ-Valerobetaine Involves PINK1/Parkin Dependent-Mitophagy and SIRT3
Source: Int J Mol Sci. 2021 Jul 29;22(15):8117. doi: 10.3390/ijms22158117 (PMC8348679; doi:10.3390/ijms22158117)
Supplement: Supplementary file 1 [file ijms-22-08117-s001.zip › ijms-1305377-supplementary.pdf]

Colorectal Cancer Apoptosis Induced by Dietary δ-Valerobetaine Involves PINK1/Parkin Dependent-Mitophagy and SIRT3

SUPPLEMENTARY FIGURE

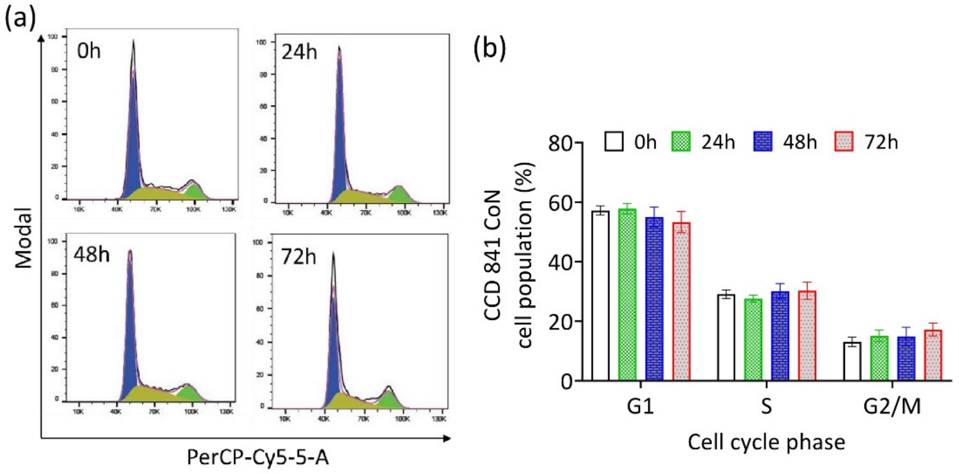

Supplementary Figure S1. Effects of δVB on normal colon cell cycle. (a) Representative cell cycle distribution and (b) analysis of CCD 841 CoN normal colon cell line. CCD 841 CoN cells were treated with 1.5 mM δVB for 24, 48 and 72 h. Cell distribution was assessed by flow cytometry collecting PI fluorescence as FL3-A (linear scale). For each sample at least 10,000 events were registered and analysis performed by using ModFIT software.

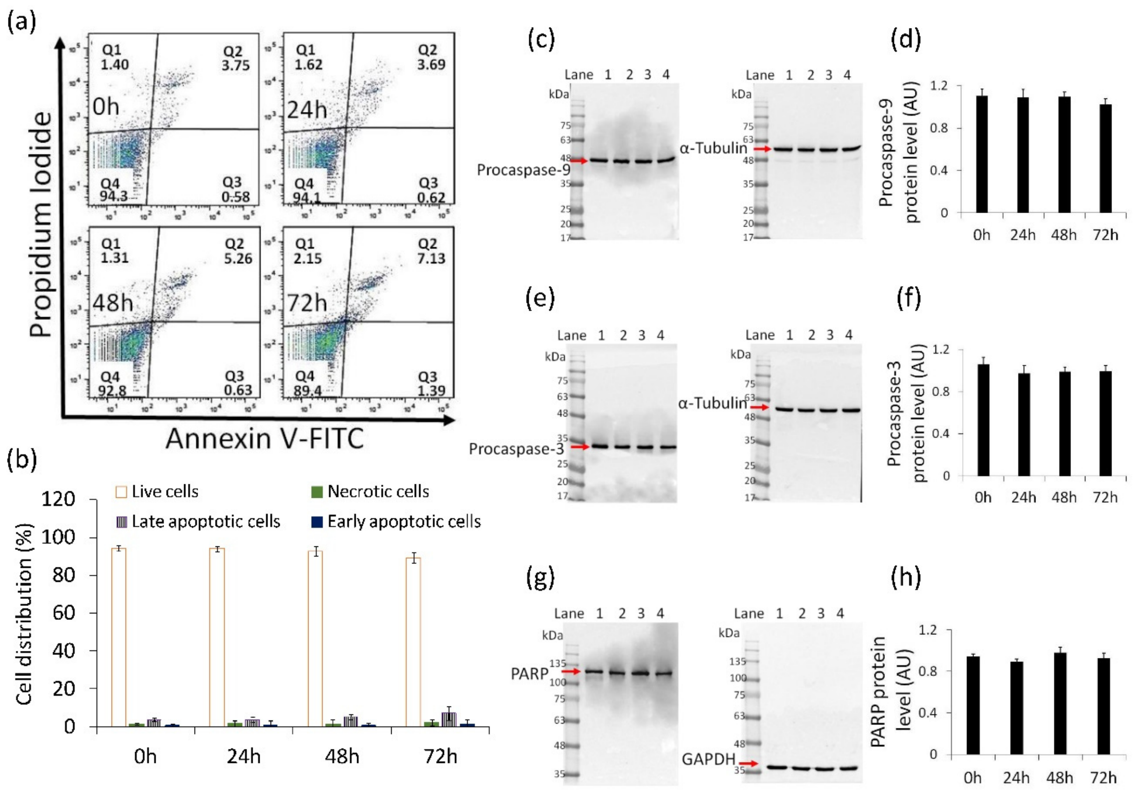

Supplementary Figure S2. Effects of δVB on normal colon cell death. (a) Representative dot plots and (b) analysis of annexin V-FITC and PI-stained CCD 841 CoN normal colon cell line. CCD 841 CoN cells were treated with 1.5 mM δVB for 24, 48 and 72 h. Cell viability/death was assessed by flow cytometry where at least 10,000 events were acquired. Q1: necrotic cells; Q2: late apoptotic cells; Q3: early apoptotic cells; Q4: viable cells. Data are expressed as mean±SD of n = 3 experiments. Representative full-length blots of Western blotting analysis of procaspase-9 (c,d), procaspase-3 (e,f) and PARP (g,h) in CCD 841 CoN cells. Lane 1 = 0 h; lane 2 = 24 h, lane 3 = 48 h, lane 4 = 72 h. Before lane 1; molecular weight markers (G266, Applied Biological Materials Inc., Richmond, BC, Canada). Protein expression was calculated, after normalization with internal control (α-tubulin or GAPDH), with Image J software and results expressed as arbitrary units (AU).
